# Supplementary material for: Epigenetic control of IL-23 expression in keratinocytes is important for chronic skin inflammation
Source: Nat Commun. 2018 Apr 12;9:1420. doi: 10.1038/s41467-018-03704-z (PMC5897363; doi:10.1038/s41467-018-03704-z)
Supplement: Supplementary file 1 — Supplementary Information(PDF 14016 kb) [file 41467_2018_3704_MOESM1_ESM.pdf]

Supplementary Information to

Li et al.: " **Epigenetic control of IL-23 expression in keratinocytes is important for chronic skin inflammation"**

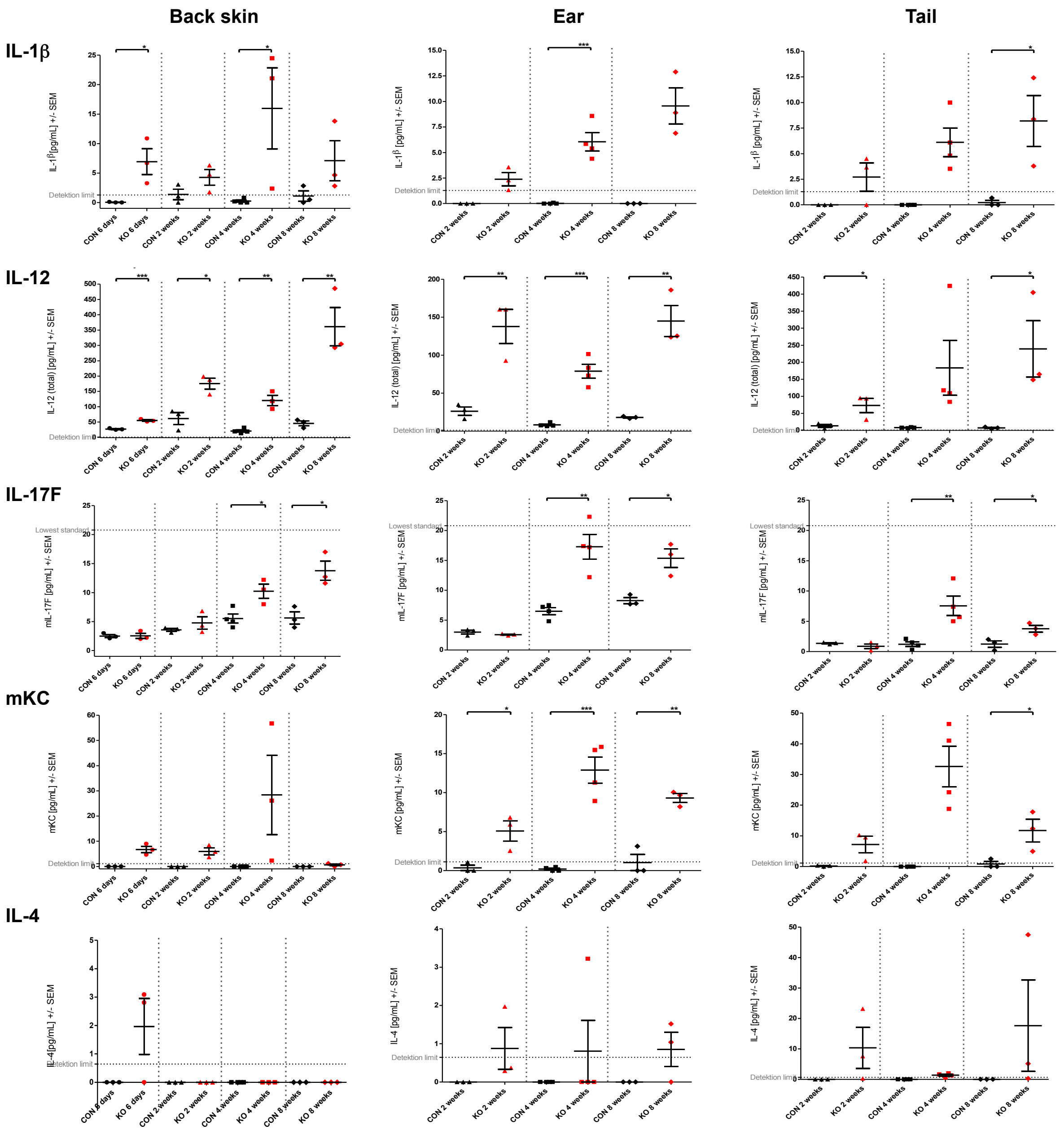

Supplementary Figure 1. Increased protein amounts of IL-12, IL-1b, mKC, IL-4 and IL-17F in N-WASP ko skin.

IL-1b, IL-12, IL-17F, mKC and IL-4 protein amounts were determined by ELISA in back skin, ear, and tail of control (black symbols) and N-WASP mutant mice (red symbols) at the indicated ages as described in “Experimental Procedures”. Shown are the averages with the standard error of means (SEM). Significance was calculated by either one-tailed t-test or one sample one-tailed t-test (the latter one was used when the expression of target protein was found undetectable in control mice) using GraphPad Prism v. 5.0 (GraphPad, San Diego, CA). (*: p<=0.05; **: p<=0.01; ***: p<=0.001).

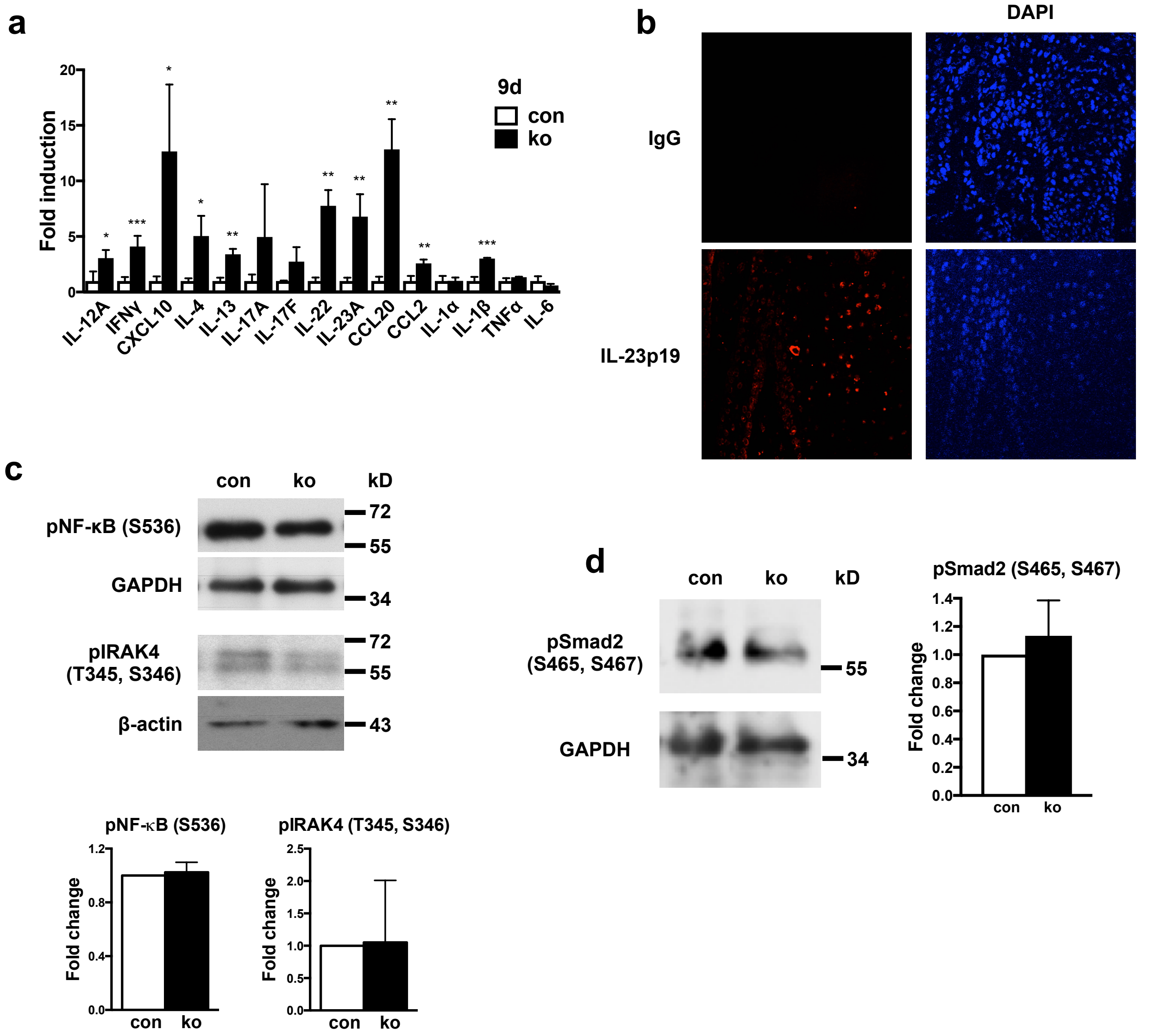

Supplementary Figure 2. Cytokines are induced in back skin of 9d old N-WASP mutant mice.
(a) Increased cytokine expression in back skin of 9d old N-WASP ko compared to control litter mates (fl/fl), as determined by qRT-PCR. All normalized to GAPDH (n>=3/3, mean +/- SD, two tailed unpaired t-test).
(b) Psoriatic skin lesioned stained for IL-23p19 and in parallel with murine IgG (negative control). DAPI counterstaining indicates epidermis (n: 2).
(c) Representative immunoblots and quantification of indicated protein modifications from epidermal lysates of 6d old con and ko mice (n: 3/3, mean +/- SD, two tailed unpaired t-test).
(d) Representative immunoblots and quantification of pSmad2 (S465, S467) from epidermal lysates of 9d old con and ko mice (n: 3/3, mean +/- SD, two tailed unpaired t-test).
(*: p<=0.05; **: p<=0.01; ***: p<=0.001).

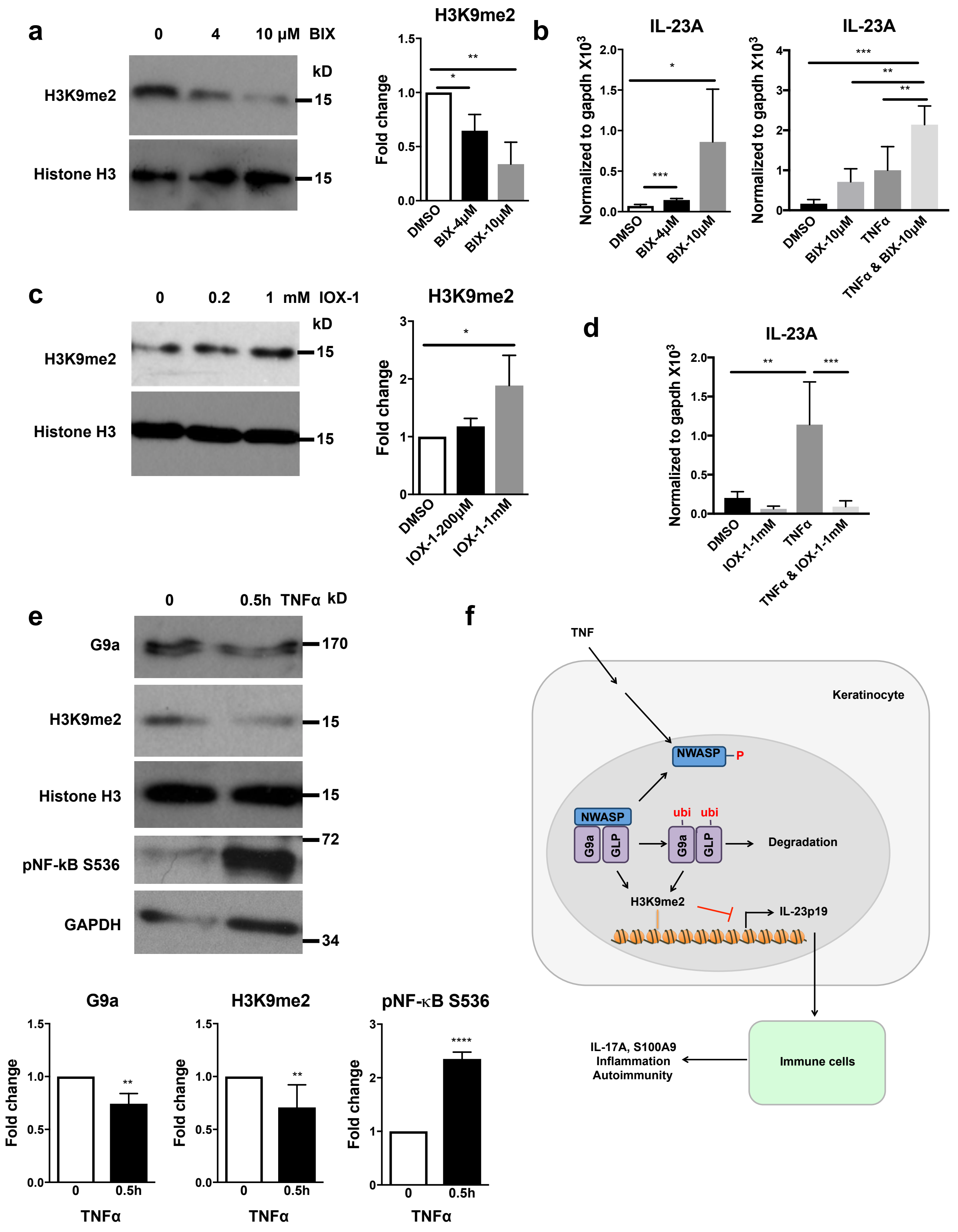

a
0 4 10 µM BIX
kD
H3K9me2
15
Histone H3
15
H3K9me2
Fold change
DMSO
BIX-4µM
BIX-10µM
b
IL-23A
Normalized to gapdh X10^3
DMSO
BIX-4µM
BIX-10µM
IL-23A
Normalized to gapdh X10^3
DMSO
BIX-10µM
TNFα
TNFα & BIX-10µM
c
0 0.2 1 mM IOX-1
kD
H3K9me2
15
Histone H3
15
H3K9me2
Fold change
DMSO
IOX-1-200µM
IOX-1-1mM
d
IL-23A
Normalized to gapdh X10^3
DMSO
IOX-1-1mM
TNFα
TNFα & IOX-1-1mM
e
0 0.5h TNFα kD
G9a
170
H3K9me2
15
Histone H3
15
pNF-kB S536
72
GAPDH
34
G9a
H3K9me2
pNF-κB S536
Fold change
0 0.5h
TNFα
f
TNF
Keratinocyte
NWASP
P
NWASP
G9a
GLP
ubi
ubi
Degradation
H3K9me2
IL-23p19
IL-17A, S100A9
Inflammation
Autoimmunity
Immune cells

Supplementary Figure 3. TNF induces IL-23p19 expression in human keratinocytes via regulation of H3K9 dimethylation.

(a) Representative immunoblots and quantification of indicated protein modifications from primary human keratinocytes treated with BIX at indicated concentrations for 24h. Quantification were normalized to histone 3 (n: 3/3, mean +/- SD, two tailed unpaired t-test).

(b) qRT-PCR analysis of IL-23A mRNA expression in primary human keratinocytes pretreated with or without BIX at indicated concentrations for 24h (left) and then stimulated with or without 10 ng ml^{-1} TNFa for 4h as indicated (right). All data are normalized to GAPDH (n: 3/3, mean +/- SD, two tailed unpaired t-test).

(c) Representative immunoblots and quantification of indicated protein modifications in primary human keratinocytes treated with IOX-1 at indicated concentrations for 24h (n: 3/3, mean +/- SD, two tailed unpaired t-test).

(d) qRT-PCR analysis of IL-23A mRNA expression in primary human keratinocytes pretreated with or without IOX-1 at indicated concentrations for 24h and then stimulated with or without 10 ngml-1 TNFa for 4h as indicated. All normalized to GAPDH (n: 3/3, mean +/- SD, two tailed unpaired t-test).

(e) Representative immunoblots and quantification of indicated protein modifications from human primary keratinocytes treated with 10 ng ml^{-1} TNFa for 0.5h (n: 3/3 for G9a, pNF-κB S536; n: 6/6 for H3K9me2, mean +/- SD, two tailed unpaired t-test).

(f) Hypothetical scheme for epigenetic control of IL-23 expression in keratinocytes. TNF stimulation leads to phosphorylation of nuclear N-WASP, which then dissociated from G9a/GLP, promoting ubiquitination and degradation of these histone methyltransferases. This results in decreased H3K9me2, which promotes IL-23 expression by keratinocytes that triggers expression of IL-17A, S100A9, skin inflammation and autoimmunity.

(*: $p<=0.05$; **: $p<=0.01$; ***: $p<=0.001$; ****: $p<=0.0001$).

Fig. 2a IgG

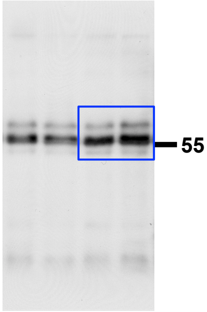

Fig.2a IgG

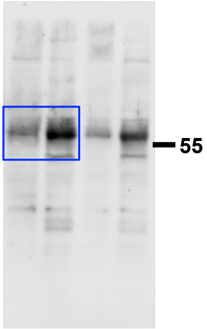

Fig.3e IL-23p19

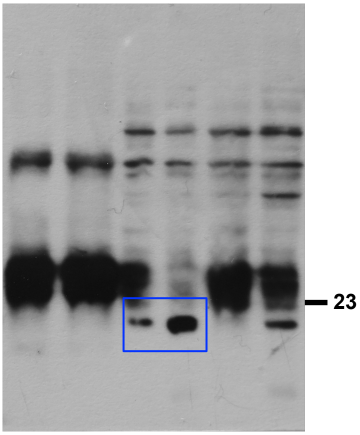

Fig.3e IL-12p40

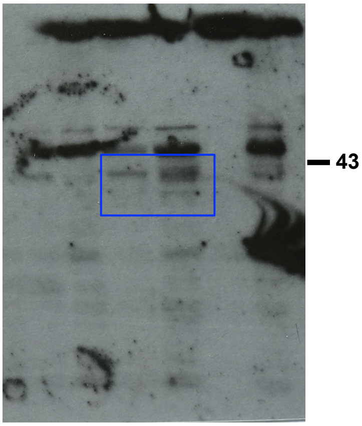

Fig.2a GAPDH

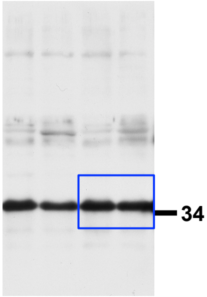

Fig.2a GAPDH

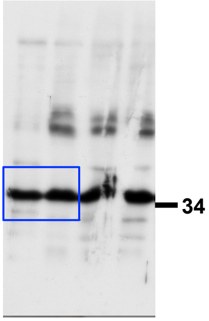

Fig.3e GAPDH

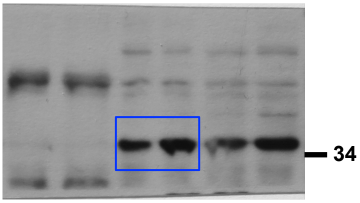

Fig.3e GAPDH

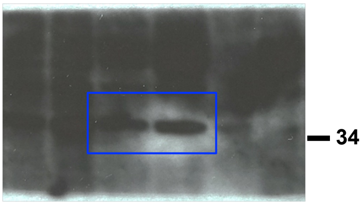

Fig.3f IL-12p40

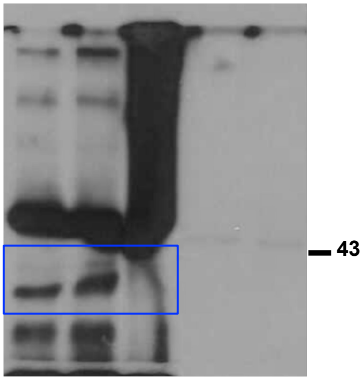

Fig. 7a H3K9me2

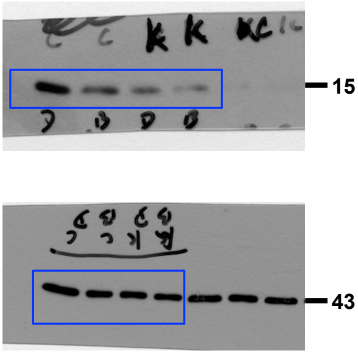

Fig. 7b H3K9me2

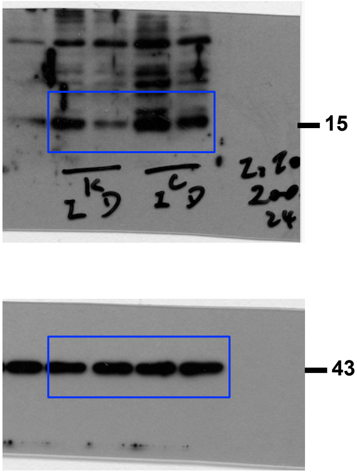

Fig.3f IL-23p19

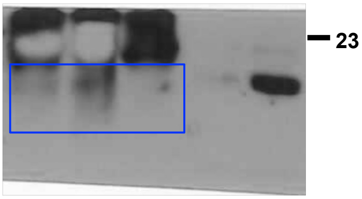

Fig. 7c H3K9me2

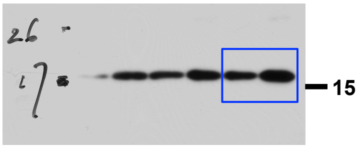

Fig. 7c GAPDH

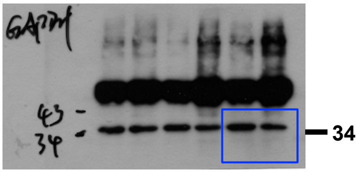

Fig. 7c N-WASP

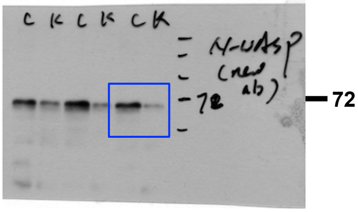

Fig. 8a N-WASP

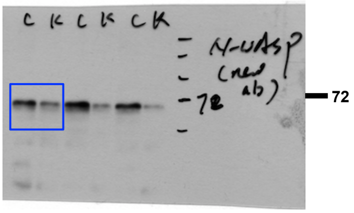

Fig. 8a G9a

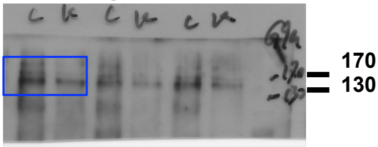

Fig. 8a GLP

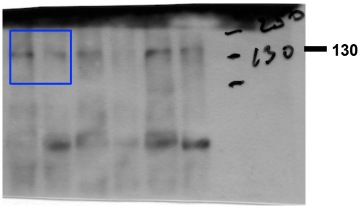

Fig. 8a GAPDH

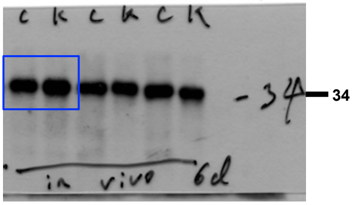

Fig. 7g N-WASP

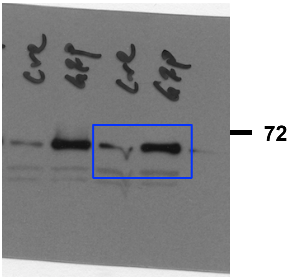

Fig. 7g β-actin

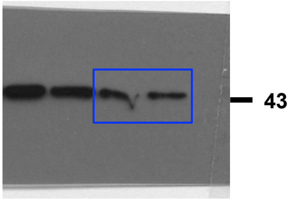

Fig. 7g G9a

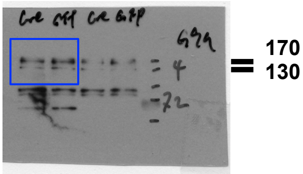

Fig. 7g H3K9me2

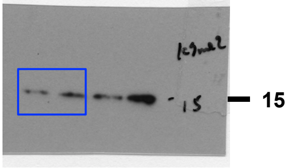

Fig. 7g β-actin

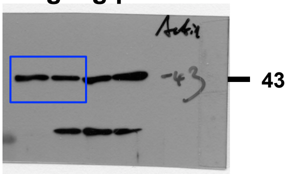

Fig. 8d G9a

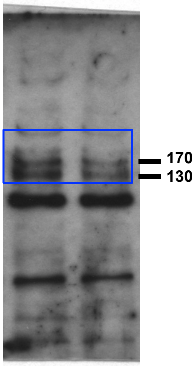

Fig. 8d GLP

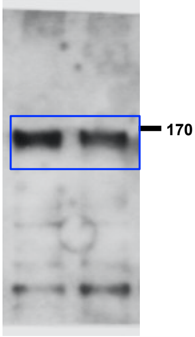

Fig. 8d N-WASP

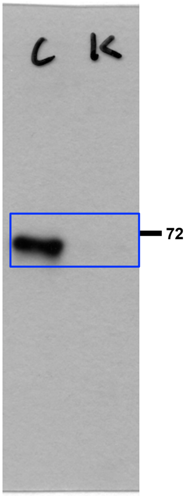

Fig. 8d GAPDH

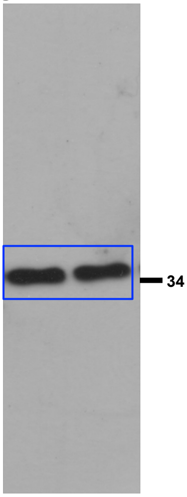

Fig. 8e G9a

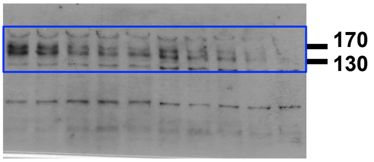

Fig. 8e GLP

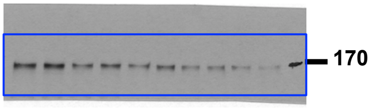

Fig. 8e N-WASP

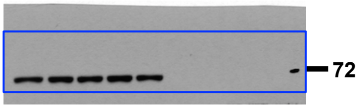

Fig. 8e GAPDH

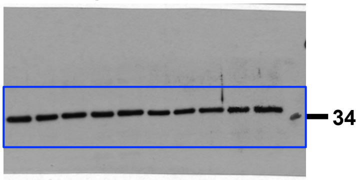

Fig. 8f N-WASP

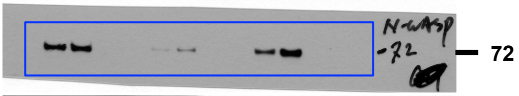

Fig. 8f G9a

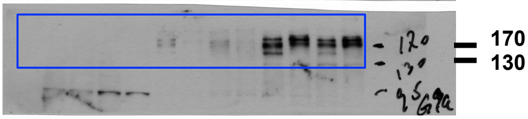

Fig. 8f GLP

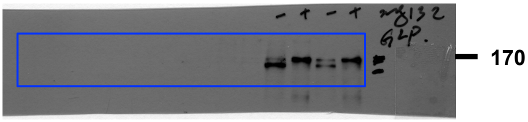

Fig. 8f H3K9me2

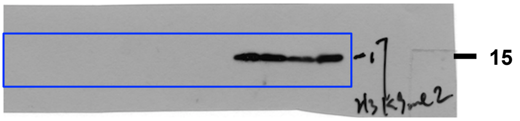

Fig. 8f Histone H3

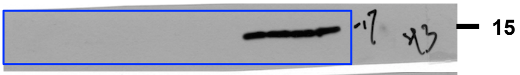

Fig. 8f PCNA

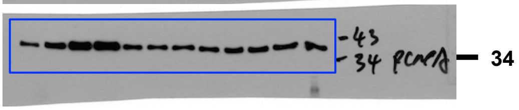

Fig. 8f GAPDH

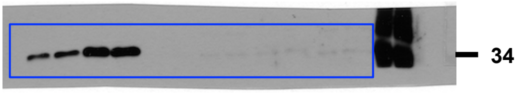

Fig. 8g N-WASP

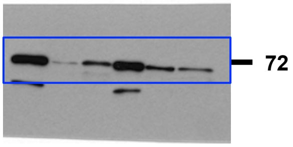

Fig. 8g G9a

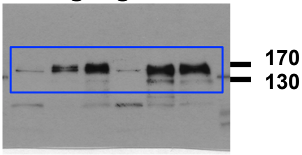

Fig. 8g GLP

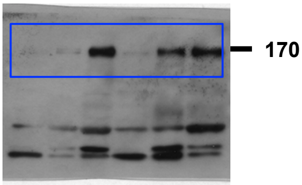

Fig. 8g Histone H3

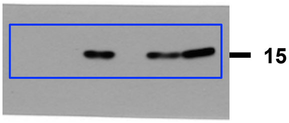

Fig. 8g PCNA

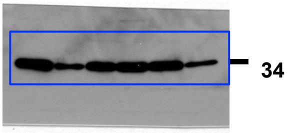

Fig. 8g GAPDH

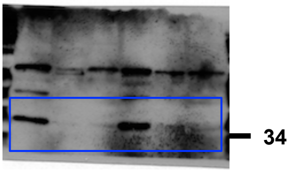

Fig. 8h G9a

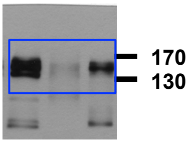

Fig. 8h GLP

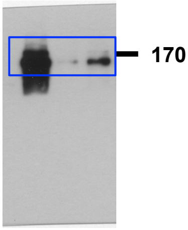

Fig. 8h N-WASP

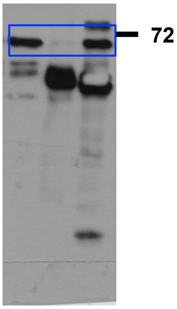

Fig. 8h G9a

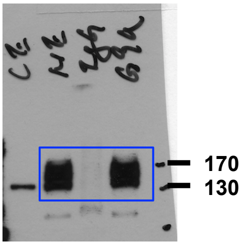

Fig. 8h GLP

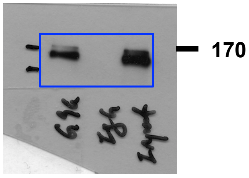

Fig. 8h N-WASP

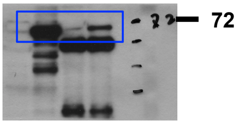

Fig. 9c pp38

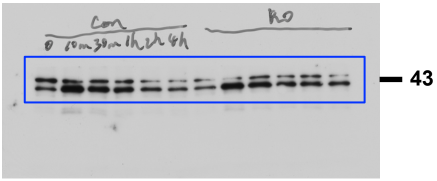

Fig. 9c pJNK

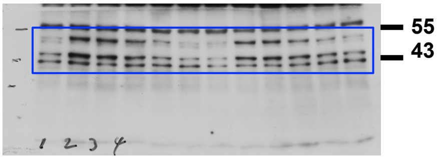

Fig. 9c pNF-κB S536

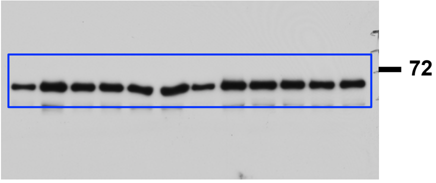

Fig. 9c G9a

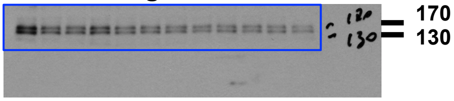

Fig. 9c GLP

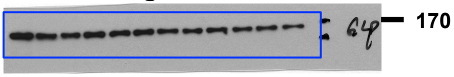

Fig. 9c N-WASP

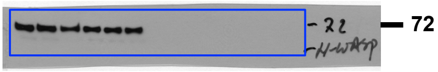

Fig. 9c GAPDH

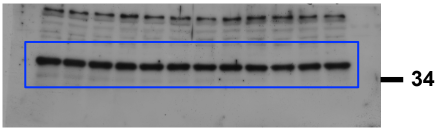

Fig. 9c H3K9me2

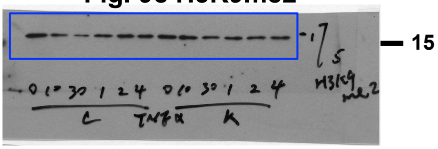

Fig. 9c Histone H3

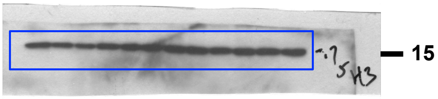

Fig. 9d N-WASP

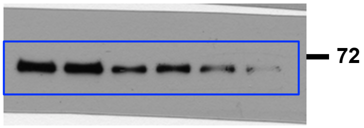

Fig. 9d N-WASP

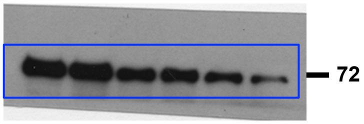

Fig. 9d pN-WASP (S480/481)

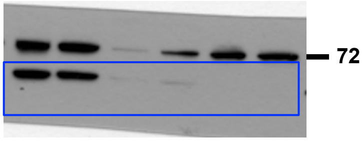

Fig. 9d pN-WASP (S480/481)

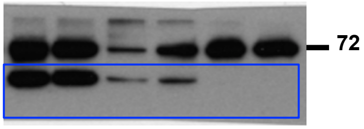

Fig. 9d PCNA

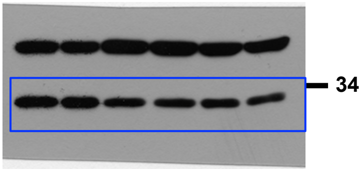

Fig. 9d GAPDH

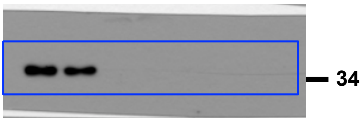

Fig. 9d Histone H3

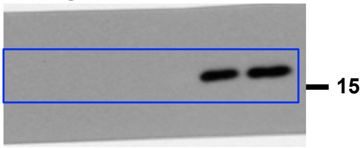

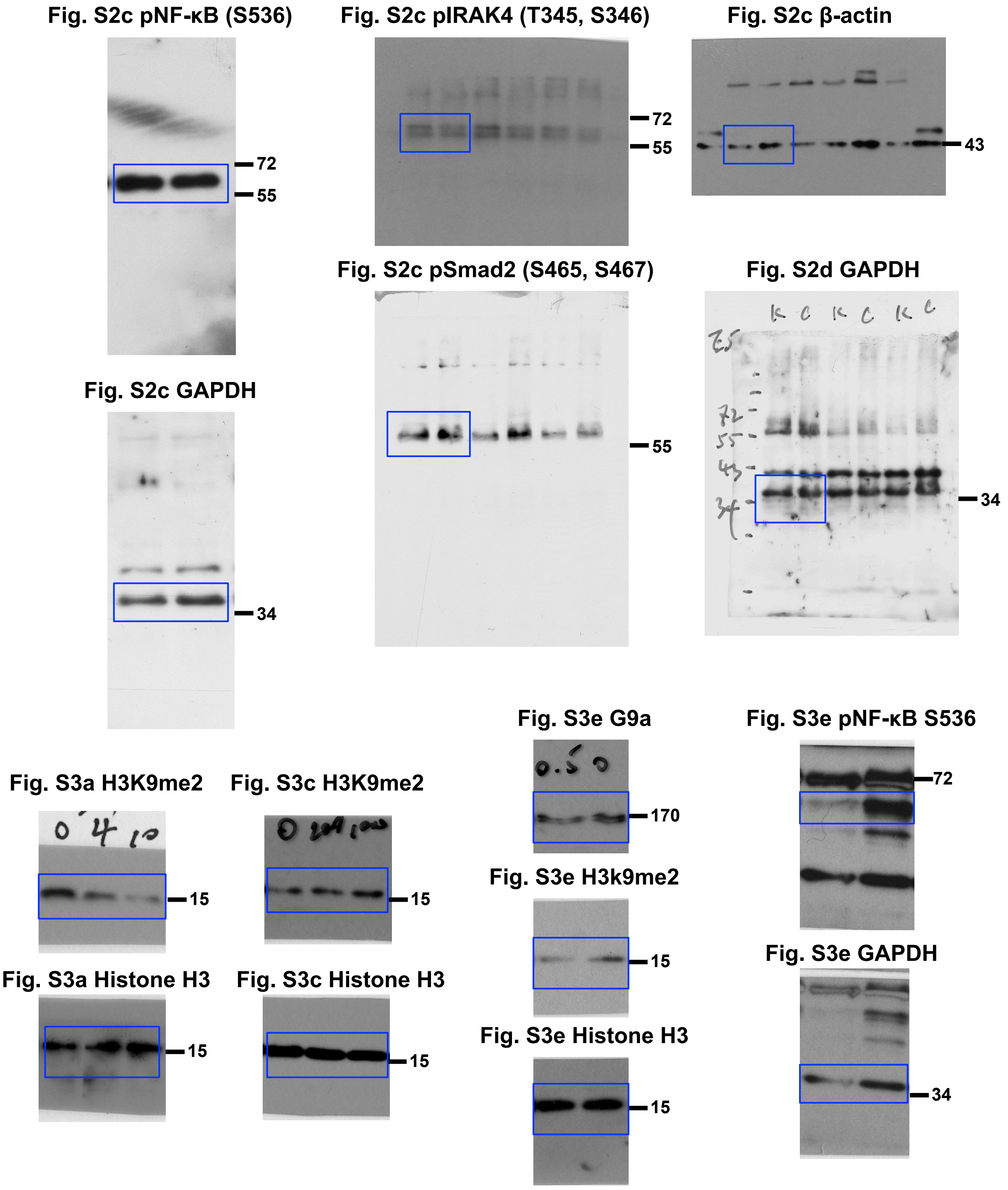

Supplementary Figure 4. Uncropped scans of blots used in the main and supplementary figures.

Supplementary Table 1

Gene name	Gene symbol	Fold change
small proline-rich protein 2H	Sprr2h	45
small proline-rich protein 1B	Sprr1b	42
late cornified envelope 3C	Lce3c	27
small proline-rich protein 2G	Sprr2g	18
small proline-rich protein 2D	Sprr2d	13
late cornified envelope 3F	Lce3f	13
small proline-rich protein 2A1	Sprr2a1	11
transglutaminase 6	Tgm6	10
transglutaminase 3. E polypeptide	Tgm3	8.9
late cornified envelope 1G	Lce1g	6.6
small proline-rich protein 2E	Sprr2e	4.8
late cornified envelope 1H	Lce1h	4.3
late cornified envelope 6A	Lce6a	4.2
small proline-rich protein 2B	Sprr2b	3.7
involucrin	Ivl	3.2
late cornified envelope 1I	Lce1i	2.9
late cornified envelope 1E	Lce1e	2.9
late cornified envelope 1K	Lce1k	2.3
small proline-rich protein 4	Sprr4	2.2
late cornified envelope 1A2	Lce1a2	2.2
cystatin A	Csta	2
late cornified envelope 1D	Lce1d	2
transglutaminase 1. K polypeptide	Tgm1	2
corneodesmosin	Cdsn	1.2
loricrin	Lor	1

Supplementary Table 2

qRT-PCR	Forward primer	Reverse primer
IL-1α	GCACCTTACACCTACCAGAGT	AAACTTCTGCCTGACGAGCTT
IL-1β	CAACCAACAAGTGATATTCTCCATG	GATCCACACTCTCCAGCTGCA
IL-4	GGTCTCAACCCCCAGCTAGT	GCCGATGATCTCTCTCAAGTGAT
IL-6	GAGGATACCACTCCCAACAGACC	AAGTGCATCATCGTTGTTCATACA
IL-12a	GGAAGCACGGCAGCAGAATA	AACTTGAGGGAGAAGTAGGAATGG
IL-12b	TGGTTTGCCATCGTTTTGCTG	ACAGGTGAGGTTCACTGTTTCT
IL-13	GGATATTGCATGGCCTCTGTAAC	AACAGTTGCTTTGTGTAGCTGA
IL-17a	TTTAACTCCCTTGGCGCAAAA	CTTTCCCTCCGCATTGACAC
IL-17f	TGCTACTGTTGATGTTGGGAC	AATGCCCTGGTTTTGGTTGAA
IL-19	CTCCTGGGCATGACGTTGATT	GCATGGCTCTCTTGATCTCGT
IL-20	TCTTGCCTTTGGACTGTTCTCC	GTTTGCAGTAATCACACAGCTTC
IL-22	ATACATCGTCAACCGCACCTTT	AGCCGGACATCTGTGTTGTTAT
IL-23a	ATGCTGGATTGCAGAGCAGTA	ACGGGGCACATTATTTTTAGTCT
IL-24	GAGCCTGCCCAACTTTTTGTG	TGTGTTGAAGAAAGGGCCAGT
CCL2	TTAAAAACCTGGATCGGAACCAA	GCATTAGCTTCAGATTTACGGGT
CCL20	CCAGGCAGAAGCAAGCAACT	TCGGCCATCTGTCTTGTGAA
CXCL5	TCCAGCTCGCCATTCATGC	TTGCGGCTATGACTGAGGAAG
CXCL10	CCAAGTGCTGCCGTCATTTTC	TCGGCCATCTGTCTTGTGAA
IFNα	GGACTTTGGATTCCCGCAGGAGAAG	GCTGCATCAGACAGCCTTGCAGGTC
IFNβ	CAGCTCCAAGAAAGGACGAAC	GGCAGTGTAACTCTTCTGCAT
IFNγ	ATGAACGCTACACACTGCATC	TCGGCCATCTGTCTTGTGAA
TNFα	CATCTTCTCAAAATTCGAGTGACAA	TCGGCCATCTGTCTTGTGAA
S100A8	AAATCACCATGCCCTCTACAAG	CCCACTTTTATCACCATCGCAA
S100A9	ATACTCTAGGAAGGAAGGACACC	TCCATGATGTCATTTATGAGGGC
LL37	GCTGTGGCGGTCACTATCAC	TGTCTAGGGACTGCTGGTTGA
G9a	GAAGTCGAAGCTCTAGCTGAAC	TGAGGAACCCACACCATTCAC
GLP	CAGATGGAGAAACAAATGGGTCT	TTTGCTTCCCCACTTCTGTGT
GAPDH	AGGTCGGTGTGAACGGATTTG	TCGGCCATCTGTCTTGTGAA
IL-23a Human	CTCAGGGACAACAGTCAGTTC	ACAGGGCTATCAGGGAGCA
GAPDH Human	GGAGCGAGATCCCTCCAAAAT	GGCTGTTGTCATACTTCTCATGG

IL-23 ChIP-qPCR	Forward primer	Reverse primer
A	TGTGCCTCCAACTCACTCTG	CTCTCTTTTGCTACATCTGCTCA
B	ACCCGGGGAATGCCCTTACTTACTATTTCT	TCAAGGTTTATTCTTACCCAACCCCAGTC
C	CAAAAGGAAGAAATCAGGAAGC	ATCTGGCTGGCTCTGTGA

CRISPR	sgRNA
IL-23 loxP1-sgRNA	GCGGTTGAGCGGAATGCAAA
IL-23 loxP2-sgRNA	CTGCCGAATATGCTCGGTCT

Homologous arm	HR-loxP1	HR- loxP2
	GCCATCGTGGAATGAGATAGGACAGAAGA<br>CTGGGGCTTCTGGAAGAGTTGTGGGCCGG<br>CG***ATAACTTCGTATAATGTATGCTATACGAA<br>GTTATGTTGAGCGGAATGCAAAGCGGTCAC<br>CTCGCCTCACTGTTCCCACTCCCTCCATTAC<br>AGAA***	GGGTTCTGAATGGACACACCTGTGTCGAG<br>TTCACTGAATGATAATACGGTATCTCCACTG<br>***ATAACTTCGTATAATGTATGCTATACGAAGT<br>TATCCGAATATGCTCGGTCTTTTAGACTTG<br>TATGTATGATGTTCATGTATGTGTATGTATAT***

Genotyping IL-23a	Forward primer	Reverse primer
IL-23-flox1	GGTTTAATGGAAGCTGTGGC	CATCTTCACACTGGATACGG
IL-23-flox2	ACAGATGGGAAAAGTAGGGC	TACATACACATACATGAACATC

Supplementary discussion

The TNF/IL-23/IL-17 cytokine cascade is suggested to be important for different inflammatory diseases. In psoriasis, inhibitors against these cytokines are already used in the therapy of this chronic skin inflammation[1, 2]. Here we show in a mouse model that keratinocytes can produce IL-23 at levels sufficient to cause differentiation of IL-17A producing T cells and skin inflammation. We furthermore describe a TNF/N-WASP/G9a, GLP/H3K9me2 pathway that crucially regulates IL-23 expression in human and mouse keratinocytes (Suppl. Fig. 3f).

These data suggest that also other signaling pathways affecting H3K9me2 could regulate IL-23 expression in keratinocytes. For example, DNA damage response (DDR) signaling induces degradation of G9a in senescent cells involving reactive oxygen species[3], which might promote IL-23 expression. Very recently, it was shown that TLR signaling prevents proteolytic degradation of the H3K9 demethylase Jmj2d in dendritic cells, resulting in decreased H3K9 methylation and increased IL-23 expression[4]. Indeed, treatment of human and mouse keratinocytes in vitro with polyIC is inducing IL-23 expression corresponding to decreased H3K9me2 (Li and Brakebusch, unpublished results). Together these findings show suggest that different signaling pathways can converge on the H3K9me2 dependent epigenetic control of IL-23 expression.

Could this mechanism be involved in the initiation or maintenance of inflammatory diseases? Mice lacking N-WASP in keratinocytes show a phenotype with partial similarities to different human diseases, suggesting that keratinocyte-derived IL-23 might contribute to certain illnesses, but is not sufficient to elicit them on its own. Differences in immune cells present in murine and human skin, however, should be taken into consideration when discussing similarities of skin disease phenotypes in human and mice.

Similar to psoriasis, N-WASP ko mice show hyperplasia, chronic inflammation, an IL-23/IL-17 profile with disease dependency on IL-23, increased expression of antimicrobial peptides such as

S100A9 or LL37, increased expression of barrier related genes, and decreased H3K9me2 and increased IL-23 expression in keratinocytes. However, other aspects such as a strong lymphocytic infiltrate are lacking. On the contrary, autoantibodies or IgG deposition in kidney as detected in N-WASP ko mice are hardly observed in psoriasis.

For the initiation of psoriasis, genetic predisposition and environmental stress factors such as wounding or streptococcal infection are crucial[5]. By which molecular mechanisms these stress factors trigger chronic skin inflammation is less clear. Our data suggest that stress-related H3K9me2 regulating pathways such as TNF, polyIC, or DDR might promote IL-23 expression in keratinocytes, which could be involved in the initiation of psoriasis. The relative contribution of keratinocytes and immune cells to the pathogenesis of psoriasis is still an open question. Our results indicate that IL-23 production by keratinocytes probably cannot be neglected and that even small relative changes can be of major importance for the development of chronic skin inflammation due to the high number of keratinocytes relative to the IL-23 producing immune cells. Crucial effectors of IL-23 are conceivably IL-17A and S100A9, which both are significantly reduced in the absence of IL-23p19 correlating with decreased inflammation. S100A9 was shown to be induced by IL-17A[6], is highly expressed in psoriasis, and deletion of the S100A9 gene in mice strongly reduces skin inflammation in various model, suggesting a strong pro-inflammatory role[7].

Although antibodies against IL-23 are currently only approved for treatment of psoriasis, IL-23 is also believed to play an important role in AD and SLE, which both display increased serum levels of IL-23[8, 9]. Indeed, many genes increased in N-WASP ko mice are also elevated in AD. Recently, injection of IL-23 in mouse skin, previously characterized as psoriasis model[10, 11], was shown to result in a gene expression profile more similar to AD than psoriasis[12]. Another very recent study in mice indirectly suggested that IL-23 produced by keratinocytes contributes to the AD phenotype[13].

Interestingly, the inflammation expression signature of mild intrinsic AD (no increased serum IgE) was reported to be more similar to psoriasis than to mild extrinsic AD (increased serum IgE[14]). Molecular pathways underlying AD and psoriasis, therefore, might be partially overlapping. Presence of autoantibodies against dsDNA as observed in the N-WASP ko mice and antibody deposition in different organs including the kidneys is highly characteristic of SLE, and not observed in AD or psoriasis. Interestingly, patients with concomitant SLE and psoriasis have been described[15], suggesting a potential relationship of both diseases via the IL-23/IL17 axis. This notion was supported by report of successful therapy of such patients with antibodies against IL-23, reducing symptoms of both SLE and psoriasis[16]. It will be interesting to test if the N-WASP/H3K9me2/IL-23 pathway and the disease relevant production of IL-23 by epithelial cells described in our study will be of importance for IL-23 related diseases such as AD and SLE.

N-WASP is a known regulator of cytoplasmic actin polymerization and was furthermore shown to regulate gene expression in an actin polymerization dependent manner by complex formation with RNA polymerase II[17]. We describe now a nuclear function for N-WASP that is probably not dependent on actin polymerization, since direct inhibition of actin polymerization decreased IL-23 expression. Yet, TNF-induced phosphorylation of N-WASP at serine 480/481 is not only decreasing complex formation with G9a and GLP, but also the ability to promote actin polymerization[18], which might indicate a link between these two processes. Actin polymerization by chromatin-bound N-WASP might furthermore facilitate changes in chromatin organization. Interestingly, it has been reported in HTC75 cells that chromatin-bound G9a is moving from a peripheral to a more central nuclear localization during mitosis[19]. G9a attached N-WASP could be involved in that movement.

It was shown earlier that opening the N-WASP conformation by binding of Cdc42-GTP strongly promoted nuclear localization[20], indicating that other pathways than TNF signaling might control nuclear function of N-WASP by regulation of its intracellular localization. Since N-WASP is linked to actin polymerization and mechanosensing, there is a possibility that N-WASP might be involved in the translation of mechanical stress to altered gene expression[21, 22]. It will be important to elucidate the crosstalk between cytoplasmic activation of N-WASP and its nuclear function and to investigate the biological importance of any coordinated activities.

CKII was shown to phosphorylate N-WASP at serine 480/481[18], but how TNF could activate CKII is not clear. Comparing this phosphorylation site with consensus phosphorylation sites of other protein kinases revealed a similarity to the PKC consensus site. Interestingly, PKCα is activated by TNF and translocated to the nucleus.

Phosphoproteomic analyses indicated multiple phosphorylation sites on N-WASP (www.phosphosite.org), hinting the possibility that also other phosphorylation sites might be involved in the regulation of the epigenetic function of N-WASP[23, 24, 25]. Previously it was demonstrated that phosphorylation of N-WASP at tyrosine 256 decreases nuclear localization[20], suggesting that also tyrosine phosphorylation of N-WASP could regulate chromatin association. Future studies will reveal domains and amino acids required for the interaction of N-WASP with G9a and GLP and the nature of the kinases involved in phosphorylation of N-WASP at serine 480/481.

In conclusion, we describe here a nuclear mechanism for TNF/N-WASP dependent epigenetic regulation of IL-23 that could explain how environmental stress affects IL-23 expression, thus contributing to chronic inflammatory diseases dependent on the IL-23/IL-17 axis.

References (In extended discussion: 2,3, 32-)

1. Kim J, Krueger JG. The immunopathogenesis of psoriasis. *Dermatol Clin* **33**, 13-23 (2015).

2. Campa M, Menter A. A review of emerging IL-17 inhibitors in the treatment of psoriasis focusing on preclinical through phase II studies. *Expert Opin Investig Drugs* **25**, 1337-1344 (2016).

3. Takahashi A*, et al.* DNA damage signaling triggers degradation of histone methyltransferases through APC/C(Cdh1) in senescent cells. *Mol Cell* **45**, 123-131 (2012).

4. Jin J*, et al.* Epigenetic regulation of the expression of Il12 and Il23 and autoimmune inflammation by the deubiquitinase Trabid. *Nat Immunol* **17**, 259-268 (2016).

5. Nestle FO, Kaplan DH, Barker J. Psoriasis. *N Engl J Med* **361**, 496-509 (2009).

6. Jin S*, et al.* DAMP molecules S100A9 and S100A8 activated by IL-17A and house-dust mites are increased in atopic dermatitis. *Exp Dermatol* **23**, 938-941 (2014).

7. Schonthaler HB*, et al.* S100A8-S100A9 protein complex mediates psoriasis by regulating the expression of complement factor C3. *Immunity* **39**, 1171-1181 (2013).

8. Guttman-Yassky E*, et al.* Low expression of the IL-23/Th17 pathway in atopic dermatitis compared to psoriasis. *J Immunol* **181**, 7420-7427 (2008).

9. Du J, Li ZS, Shi JW, Bi LQ. Associations between serum interleukin-23 levels and clinical characteristics in patients with systemic lupus erythematosus. *J Int Med Res* **42**, 1123-1130 (2014).

10. Chan JR*, et al.* IL-23 stimulates epidermal hyperplasia via TNF and IL-20R2-dependent mechanisms with implications for psoriasis pathogenesis. *J Exp Med* **203**, 2577-2587 (2006).

11. Swindell WR*, et al.* Genome-Wide Expression Profiling of Five Mouse Models Identifies Similarities and Differences with Human Psoriasis. *PLoS One* **6**, (2011).

12. Noda S*, et al.* Major differences between human atopic dermatitis phenotype and mouse models as determined by global genomic profiling. *Journal of Investigative Dermatology* **135**, S67-S67 (2015).

13. Yoon J*, et al.* IL-23 induced in keratinocytes by endogenous TLR4 ligands polarizes dendritic cells to drive IL-22 responses to skin immunization. *Journal of Experimental Medicine* **213**, 2147-2166 (2016).

14. Martel BC*, et al.* Distinct molecular signatures of mild extrinsic and intrinsic atopic dermatitis. *Exp Dermatol* **25**, 453-459 (2016).

15. Prieto-Barrios M*, et al.* Two poles of the Th 17-cell-mediated disease spectrum: Analysis of a case series of 21 patients with concomitant lupus erythematosus and psoriasis. *J Eur Acad Dermatol Venereol*, (2016).

16. Varada S, Gottlieb AB, Merola JF, Saraiya AR, Tintle SJ. Treatment of coexistent psoriasis and lupus erythematosus. *J Am Acad Dermatol* **72**, 253-260 (2015).

17. Wu X, Yoo Y, Okuhama NN, Tucker PW, Liu G, Guan JL. Regulation of RNA-polymerase-II-dependent transcription by N-WASP and its nuclear-binding partners. *Nat Cell Biol* **8**, 756-763 (2006).

18. Galovic M, Xu D, Areces LB, van der Kammen R, Innocenti M. Interplay between N-WASP and CK2 optimizes clathrin-mediated endocytosis of EGFR. *J Cell Sci* **124**, 2001-2012 (2011).

19. Kind J*, et al.* Single-cell dynamics of genome-nuclear lamina interactions. *Cell* **153**, 178-192 (2013).

20. Wu X, Suetsugu S, Cooper LA, Takenawa T, Guan JL. Focal adhesion kinase regulation of N-WASP subcellular localization and function. *J Biol Chem* **279**, 9565-9576 (2004).

21. Spinardi L*, et al.* A dynamic podosome-like structure of epithelial cells. *Exp Cell Res* **295**, 360-374 (2004).

22. Albiges-Rizo C, Destaing O, Fourcade B, Planus E, Block MR. Actin machinery and mechanosensitivity in invadopodia, podosomes and focal adhesions. *J Cell Sci* **122**, 3037-3049 (2009).

23. Christensen GL*, et al.* Quantitative phosphoproteomics dissection of seven-transmembrane receptor signaling using full and biased agonists. *Mol Cell Proteomics* **9**, 1540-1553 (2010).

24. Olsen JV*, et al.* Quantitative phosphoproteomics reveals widespread full phosphorylation site occupancy during mitosis. *Sci Signal* **3**, ra3 (2010).

25. Goswami T*, et al.* Comparative phosphoproteomic analysis of neonatal and adult murine brain. *Proteomics* **12**, 2185-2189 (2012).
